# Molecular detection and phylogenetic characterization of *Ehrlichia*- and *Anaplasma*-related genotypes in free-ranging anteaters from southeastern Brazil

**DOI:** 10.1007/s11259-026-11375-1

**Published:** 2026-06-30

**Authors:** João Otávio Mochiuti, Mateus Oliveira Machado, Lígia Souza Lima Silveira da Mota

**Affiliations:** 1 Institute of Biosciences, Department of Genetics, Microbiology and Immunology, São Paulo State University (UNESP), Botucatu, SP Brazil; 2 School of Veterinary Medicine and Animal Science, Graduate Program in Wild Animals, São Paulo State University (UNESP), Botucatu, SP Brazil

**Keywords:** *Myrmecophagidae*, wildlife pathogens, molecular epidemiology, phylogenetic inference, vector-borne bacteria, conservation medicine

## Abstract

Bacteria of the family *Anaplasmataceae* are tick-borne pathogens of recognized veterinary and zoonotic relevance, widely distributed among domestic and wild vertebrate hosts. Although increasingly reported in Brazilian wildlife species, molecular data on these agents in xenarthrans remain scarce, particularly in regions undergoing environmental transformation. This study investigated the occurrence of *Anaplasma* spp. and *Ehrlichia* spp. in free-ranging giant anteaters (*Myrmecophaga tridactyla*) and southern tamanduas (*Tamandua tetradactyla*) from the central-western region of São Paulo State, Brazil. Twenty-six blood samples (21 *M. tridactyla* and 5 *T. tetradactyla*) were analyzed using conventional and nested PCR assays targeting the 23 S rRNA gene (*Anaplasma* spp.) and the *dsb* gene (*Ehrlichia* spp.), followed by sequencing and phylogenetic inference. An *Ehrlichia* genotype phylogenetically related to *Ehrlichia chaffeensis* was detected in two *M. tridactyla* samples (7.69%), whereas an *Anaplasma* genotype was detected in one *T. tetradactyla* sample (3.85%). The *Ehrlichia* sequences showed high nucleotide identity (99.61–100%) with reference sequences identified as *E. chaffeensis* and grouped within clades containing *E. chaffeensis* in Maximum Likelihood analyses. The *Anaplasma* sequence showed phylogenetic affinity with a clade comprising *Anaplasma marginale*, *Anaplasma centrale*, and related genotypes. Because only partial gene fragments were analyzed, species-level assignment was not attempted. These findings provide molecular evidence of the occurrence of *Anaplasmataceae*-related genotypes in free-ranging xenarthrans inhabiting anthropogenically altered landscapes in southeastern Brazil. Further investigations including expanded sampling, vector identification, and multilocus or genomic approaches are necessary to better characterize these agents and clarify their ecological significance.

## Introduction

Bacteria of the family *Anaplasmataceae* comprise obligate intracellular, tick-borne pathogens of recognized veterinary and zoonotic relevance. Species within the genera *Anaplasma* and *Ehrlichia* infect a wide range of domestic and wild vertebrate hosts, and several are associated with emerging infectious diseases of public health importance (Paddock and Childs 2003; Zanella 2016).

In Brazil, molecular detection of *Ehrlichia* and *Anaplasma* species has been reported in cervids, carnivores, rodents, marsupials, birds, and captive wild mammals, highlighting the broad host spectrum of these bacteria in wildlife (Machado et al. 2006; André et al. 2010, 2012; Benevenute et al. 2017; Alabí Córdova et al. 2024). Although *Anaplasmataceae* infections have previously been reported in xenarthrans, including records from São Paulo State (Calchi et al. 2020; Sada et al. 2024), available molecular data remain limited and geographically restricted. Additional studies incorporating new localities, host species, and sequence-based characterization are needed to improve understanding of the circulation and diversity of these agents in this understudied mammalian order.

Giant anteaters (*Myrmecophaga tridactyla*), classified as Vulnerable on the IUCN Red List (Miranda et al. 2025), and southern tamanduas (*Tamandua tetradactyla*), listed as Least Concern given their wide distribution and presumed large population (Molina et al. 2025), are both widely distributed in Brazil and frequently inhabit fragmented or anthropogenically altered landscapes (Miranda et al. 2025; Molina et al. 2025). Habitat fragmentation and human-modified landscapes may increase opportunities for contact among wildlife, domestic animals, ectoparasites, and humans, potentially facilitating pathogen exchange within a One Health context (Clark et al. 2018; Machtinger et al. 2024; Damian 2025). Previous studies have documented molecular detection of hemoparasites and other vector-borne agents in anteaters, including *Trypanosoma cruzi*, *T. rangeli*, and *Leishmania infantum* (De Araujo et al. 2013; Calchi et al. 2020).

Given the ecological relevance of xenarthrans and the ongoing environmental transformation in southeastern Brazil, investigating the occurrence and genetic diversity of Anaplasmataceae in these hosts may contribute to expanding current knowledge of pathogen diversity and distribution in wildlife. Therefore, the present study aimed to investigate the occurrence of *Anaplasma* spp. and *Ehrlichia* spp. in free-ranging anteaters from the central-western region of São Paulo State, Brazil, using PCR-based detection, sequencing, and phylogenetic inference.

## Materials and methods

Blood samples (*n* = 26) obtained from the Center for Wild Animal Medicine and Research (CEMPAS), São Paulo State University (UNESP), Botucatu, São Paulo, Brazil, were analyzed, comprising 21 individuals of *Myrmecophaga tridactyla* and 5 of *Tamandua tetradactyla*. The investigated group consisted of 15 males (57.69%), 10 females (38.46%), and 1 individual of undetermined sex (3.85%). Regarding age classification, 2 animals were juveniles (7.69%), 4 were classified as young adults (15.38%), 19 as adults (73.08%), and 1 individual had undetermined age (3.85%). Information regarding sex and age class was retrieved when available from clinical records. Detailed information on clinical presentation and rescue circumstances was not consistently available for all animals and therefore was not included in subsequent analyses.

Samples originated from municipalities located in the central-western region of São Paulo State, Brazil, including Abaitinga (*n* = 1), Areiópolis (*n* = 1), Botucatu (*n* = 6), Cerqueira César (*n* = 1), Cesário Lange (*n* = 1), Conchas (*n* = 3), Coronel Macedo (*n* = 1), Itaí (*n* = 2), Itaquecetuba (*n* = 1), Jaú (*n* = 1), Jumirim (*n* = 1), Lençóis Paulista (*n* = 2), Pardinho (*n* = 1), Piratininga (*n* = 1), Promissão (*n* = 1), São Manuel (*n* = 1), and Tietê (*n* = 1).

All procedures involving animals were conducted in accordance with institutional and national ethical standards. Sampling and handling protocols were approved by the Animal Use Ethics Committee of São Paulo State University (CEUA–UNESP; protocol no. 000.051/2024) and authorized by the Brazilian Biodiversity Authorization and Information System (SISBIO; permit no. 86964-1).

Total genomic DNA was extracted using the DNeasy Blood & Tissue Kit (QIAGEN, Germany), according to the manufacturer’s instructions. DNA quality and suitability for downstream PCR amplification were assessed by amplification of the endogenous *GAPDH* gene (Birkenheuer et al. 2003).

Samples positive for *GAPDH* amplification were subsequently screened using conventional PCR targeting the 23 S rRNA gene for *Anaplasma* spp. (Dahmani et al. 2015) and nested PCR targeting the *dsb* gene for *Ehrlichia* spp. (Doyle et al. 2005; Almeida et al. 2013). Amplified products were purified and submitted to bidirectional Sanger sequencing.

Sanger chromatograms were visually inspected and manually curated prior to consensus generation. Consensus sequences were edited and assembled using Geneious version 4.8.5 and subsequently compared with reference sequences using BLASTn. The sequences generated in this study were deposited in GenBank under accession numbers PZ060335–PZ060336 (*dsb* gene; *Ehrlichia*-related genotypes) and PX496885.1 (23 S rRNA gene; *Anaplasma* genotype).

Phylogenetic analyses were performed independently for each molecular marker and corresponding bacterial genus using MEGA11 (Tamura et al. 2021) under the Maximum Likelihood method with 1,000 bootstrap replicates (Felsenstein 1985). Separate datasets were constructed for *Ehrlichia* using partial *dsb* gene sequences and for *Anaplasma* using partial 23 S rRNA gene sequences. Reference sequences were selected from GenBank considering both BLASTn similarity and targeted literature review to improve taxonomic representation, geographic context, and phylogenetic interpretability for each marker dataset. Sequence inclusion prioritized compatibility among homologous loci and representation of taxa relevant to the diversity of Anaplasmataceae. Sequence alignments were generated independently for each dataset using the MUSCLE algorithm implemented in MEGA11 under default parameters prior to phylogenetic reconstruction. The best-fitting nucleotide substitution models were selected independently for each dataset within MEGA11. The *Ehrlichia* dataset was analyzed under the Tamura 3-parameter model with Gamma distribution (+ G), whereas the *Anaplasma* dataset was analyzed under the Tamura 3-parameter model. Trees were rooted using *Anaplasma phagocytophilum* and *Ehrlichia ruminantium*, respectively. Bootstrap values are presented at the nodes of the final trees.

## Results and discussion

DNA from an *Ehrlichia*-related genotype was detected in 7.69% (2/26; 95% CI: 2.14%–23.87%) of the sampled anteaters, whereas an *Anaplasma* genotype was detected in 3.85% (1/26; 95% CI: 0.68%–18.89%). When stratified by host species, positivity was observed in 9.52% (2/21; 95% CI: 2.66%–28.73%) of *Myrmecophaga tridactyla* and in 20.00% (1/5; 95% CI: 3.62%–62.45%) of *Tamandua tetradactyla*.

**Fig. 1:**
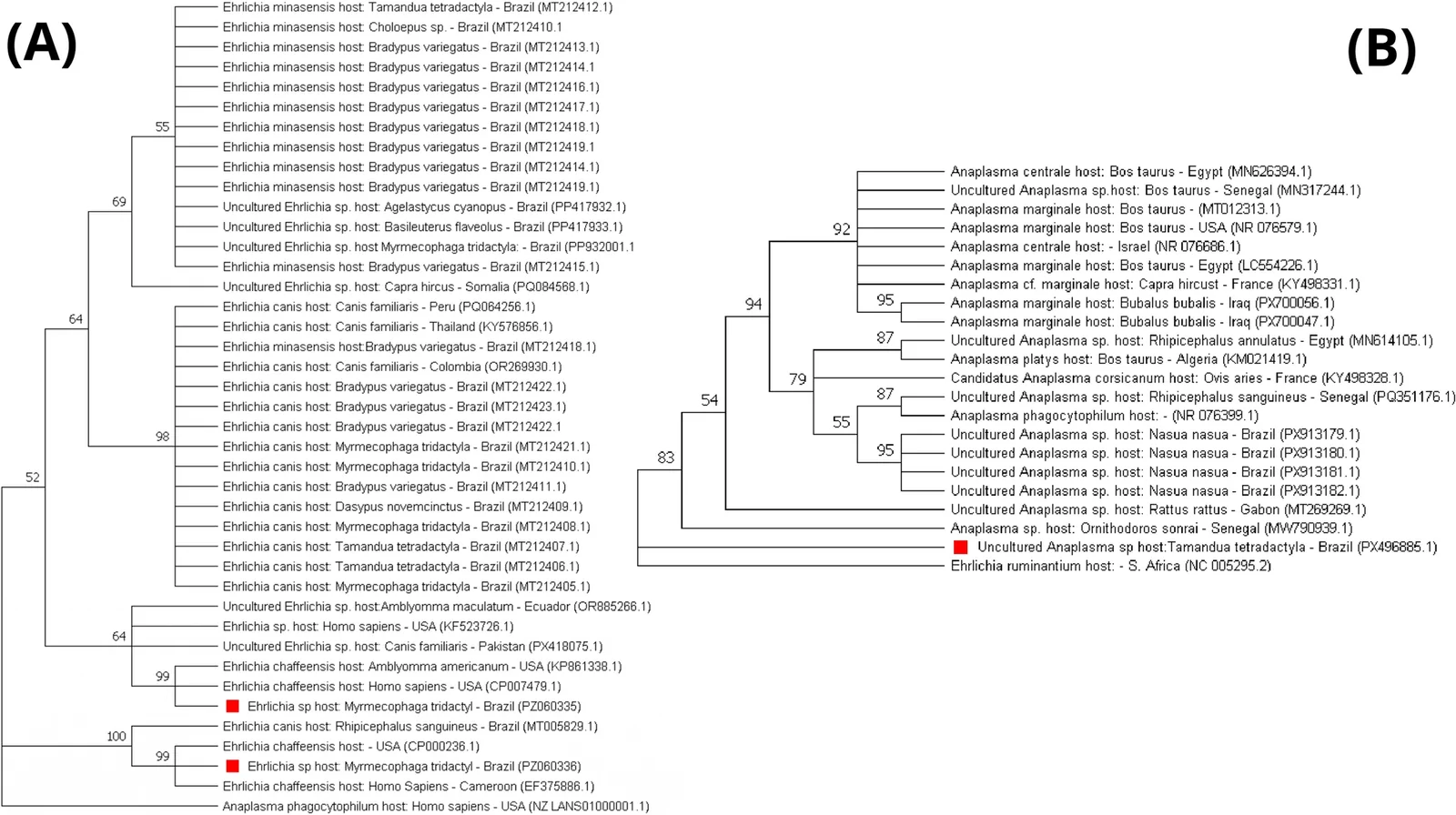
Phylogenetic analyses of *Ehrlichia*- and *Anaplasma*-related genotypes detected in free-ranging anteaters

BLASTn analysis demonstrated 100% nucleotide identity (sample 8) and 99.61% identity (sample 14) with reference sequences identified as *Ehrlichia chaffeensis*, including the Wakulla (CP007479.1) and Arkansas (CP000236.1) strains. In phylogenetic analyses, both sequences grouped within clades containing *E. chaffeensis* reference sequences (Fig. 1A). Sample PZ060335 clustered within a clade containing reference sequences identified as *E. chaffeensis*, whereas PZ060336 grouped within a broader clade containing *E. chaffeensis* and related *Ehrlichia* genotypes. Although bootstrap support was high for these groupings (bootstrap = 99), interpretation at species level should be approached cautiously because only a partial fragment of the dsb gene was analyzed. Therefore, the sequences detected in this study were conservatively interpreted as *Ehrlichia* genotypes phylogenetically related to *E. chaffeensis*. The dsb sequences generated in this study were deposited in GenBank under accession numbers PZ060335 (sample 8) and PZ060336 (sample 14).

The molecular detection of *Ehrlichia*-related genotypes in *M. tridactyla* contributes additional information regarding the occurrence of *Anaplasmataceae* in xenarthrans. Previous studies have documented infections by *Ehrlichia* and *Anaplasma* in anteaters from Brazil (Calchi et al. 2020; Sada et al. 2024), suggesting that these bacteria may circulate across distinct xenarthran hosts and geographic regions.

BLASTn analysis indicated sequence similarity with previously reported *Anaplasma* genotypes deposited in GenBank. Because sequence similarity alone does not support taxonomic assignment, BLAST results were interpreted only as complementary evidence and considered together with phylogenetic reconstruction. In Maximum Likelihood phylogenetic analysis, the sequence occupied a sister position relative to a clade comprising *Anaplasma marginale*, *Anaplasma centrale*, and related *Anaplasma* genotypes (Fig. 1B). Despite its phylogenetic proximity to these taxa, the sequence did not cluster directly within any currently recognized species-level group. Because only a partial fragment of the 23 S rRNA gene was analyzed, and because phylogenetic proximity alone does not support taxonomic assignment, the detected sequence was conservatively interpreted as an *Anaplasma* genotype. The sequence generated in this study was deposited in GenBank under accession number PX496885.1.

Similar findings involving genetically differentiated *Anaplasma* genotypes have been reported in wildlife from Brazil and elsewhere (Calchi et al. 2020), reinforcing current gaps in understanding the diversity and host associations of *Anaplasmataceae* circulating in wild mammals. However, additional loci and broader comparative datasets will be necessary to refine taxonomic interpretation and evaluate evolutionary relationships.

From an ecological perspective, the present findings indicate molecular detection of Anaplasmataceae-related genotypes in free-ranging xenarthrans inhabiting anthropogenically altered landscapes. However, detection of bacterial DNA alone does not permit inference regarding host competence, maintenance of transmission cycles, or epidemiological significance. The absence of concomitant tick collection and identification, bacterial isolation, and longitudinal monitoring further limits ecological interpretation.

The detection frequency observed in the present study should be interpreted cautiously considering the wide confidence intervals and limited number of positive individuals. Additionally, no consistent information regarding clinical condition was available for the sampled animals, preventing assessment of possible clinical or subclinical infection.

From a conservation and One Health perspective, the detection of *Anaplasmataceae*-related genotypes in free-ranging xenarthrans highlights the importance of incorporating pathogen surveillance into wildlife health monitoring programs. Future investigations including expanded geographic sampling, vector identification, and multilocus or genomic approaches will be important to better characterize these agents and clarify their ecological significance.

## Conclusion

This study provides molecular evidence of the occurrence of Anaplasmataceae-related genotypes in free-ranging anteaters from central-western São Paulo State, southeastern Brazil. *Ehrlichia* genotypes phylogenetically related to *Ehrlichia chaffeensis* were detected in *Myrmecophaga tridactyla*, whereas an *Anaplasma* genotype occupying a phylogenetic position relative to the *Anaplasma marginale–Anaplasma centrale* complex was identified in *Tamandua tetradactyla*. These findings expand current knowledge regarding the occurrence of Anaplasmataceae in xenarthrans and reinforce the value of wildlife pathogen surveillance within a One Health framework. Further investigations including expanded sampling, vector identification, and multilocus or genomic approaches will be important to improve characterization of these detected genotypes and better understand their ecological context.

## Data Availability

The nucleotide sequences generated in this study are available in GenBank under accession numbers PZ060335–PZ060336 (dsb gene, *Ehrlichia chaffeensis* ) and PX496885.1 (23 S rRNA gene, *Anaplasma* sp.). Additional information supporting the findings of this study is available from the corresponding author upon reasonable request.
